# Erratum: Dietary Patterns and Cardiovascular Risk Factors in Spanish Adolescents: A Cross-Sectional Analysis of the SI! Program for Health Promotion in Secondary Schools; *Nutrients* 2019, *11*, 2297

**DOI:** 10.3390/nu11123021

**Published:** 2019-12-10

**Authors:** Patricia Bodega, Juan Miguel Fernández-Alvira, Gloria Santos-Beneit, Amaya de Cos-Gandoy, Rodrigo Fernández-Jiménez, Luis Alberto Moreno, Mercedes de Miguel, Vanesa Carral, Xavier Orrit, Isabel Carvajal, Carolina E. Storniolo, Anna Tresserra-Rimbau, Mónica Doménech, Ramón Estruch, Rosa María Lamuela-Raventós, Valentín Fuster

**Affiliations:** 1 Foundation for Science, Health and Education (SHE), 08008 Barcelona, Spain; gsantos@fundacionshe.org (G.S.-B.); adecos@fundacionshe.org (A.d.C.-G.); mdemiguel@fundacionshe.org (M.d.M.); vcarral@fundacionshe.org (V.C.); xorrit@fundacionshe.org (X.O.); icarvajal@fundacionshe.org (I.C.); 2 Centro Nacional de Investigaciones Cardiovasculares (CNIC), 28029 Madrid, Spain; jmfernandeza@cnic.es (J.M.F.-A.); rodrigo.fernandez@cnic.es (R.F.-J.); 3 The Zena and Michael A. Wiener Cardiovascular Institute, Icahn School of Medicine at Mount Sinai, New York, NY 10029, USA; 4 Centro de Investigación Biomédica En Red en enfermedades CardioVasculares (CIBERCV), 28029 Madrid, Spain; 5 GENUD (Growth, Exercise, NUtrition and Development) Research Group, Faculty of Health Science, University of Zaragoza, 50009 Zaragoza, Spain; lmoreno@unizar.es; 6 Consorcio CIBER, M.P. Fisiopatología de la Obesidad y Nutrición (CIBERObn), Instituto de Salud Carlos III (ISCIII), 28029 Madrid, Spain; carolinastorniolo@outlook.com (C.E.S.); anna.tresserra@iispv.cat (A.T.-R.); MDOMEN@clinic.cat (M.D.); RESTRUCH@clinic.cat (R.E.); lamuela@ub.edu (R.M.L.-R.); 7 Department of Nutrition, Food Science and Gastronomy, School of Pharmacy and Food Sciences, XaRTA, INSA, University of Barcelona, 08028 Barcelona, Spain; 8 Universitat Rovira i Virgili, Departament de Bioquímica i Biotecnologia, Unitat de Nutrició Humana, Hospital Universitari Sant Joan de Reus, Institut d’Investigació Pere Virgili (IISPV), 43201 Reus, Spain; 9 Department of Internal Medicine, Hospital Clínic, Institut d’Investigacions Biomèdiques August Pi I Sunyer (IDIBAPS), University of Barcelona, 08036 Barcelona, Spain

The authors have requested that the following changes be made to their paper [1].

**Correction 1**

The following content in the Abstract on page 1: 

**“**No overall differences in CVRF were observed between clusters except for z-BMI values, total cholesterol, and non-HDL cholesterol, with the *Processed* cluster showing the lowest mean values.”

was changed to: 

**“**No overall differences in CVRF were observed between clusters except for z-BMI and z-FMI values, total cholesterol, and non-HDL cholesterol, with the *Processed* cluster showing the lowest mean values.”

**Correction 2**

The following content in the Discussion section on page 8: 

“Our analysis of associations between DPs and CVRF only found significant associations for z-BMI, TC, and non-HDL cholesterol, with z-BMI in particular being higher in the *Traditional* and *Healthy* clusters than in the *Processed* cluster.” 

was changed to: 

“Our analysis of associations between DPs and CVRF only found significant associations for z-BMI, z-FMI, TC, and non-HDL cholesterol, with z-BMI in particular being higher in the *Traditional* and *Healthy* clusters than in the *Processed* cluster.” 

**Correction 3**

The following content related to z-FMI in the Appendix section, Table A1, page 10:

**Table A1:** Cardiovascular risk factors of participants according to tertiles of principal component analysis (PCA).

	Processed				Traditional				Healthy			
	T1	T2	T3	*p* for Trend	T1	T2	T3	*p* For Trend	T1	T2	T3	*p* For Trend
	Mean * (95% CI)	Mean * (95% CI)	Mean * (95% CI)		Mean * (95% CI)	Mean * (95% CI)	Mean * (95% CI)		Mean * (95% CI)	Mean * (95% CI)	Mean * (95% CI)	
z-FMI	−0.01 (−0.05, 0.03)	0.01 (−0.03, 0.05)	−0.04 (−0.07, 0.00)	0.211	−0.01 (−0.05, 0.03)	0.00 (−0.03, 0.04)	0.03 (−0.06, 0.01)	0.528	0.00 (−0.04, 0.03)	−0.02 (−0.06, 0.02)	−0.01 (−0.05, 0.03)	0.834

was changed to: 

**Table A1:** Cardiovascular risk factors of participants according to tertiles of principal component analysis (PCA).

	Processed				Traditional				Healthy			
	T1	T2	T3	*p* for Trend	T1	T2	T3	*p* For Trend	T1	T2	T3	*p* For Trend
	Mean * (95% CI)	Mean * (95% CI)	Mean * (95%CI)		Mean * (95% CI)	Mean * (95% CI)	Mean * (95% CI)		Mean * (95% CI)	Mean * (95% CI)	Mean * (95% CI)	
z-FMI	−0.01 (−0.04, 0.07)	0.01 (−0.04, 0.07)	−0.04 (−0.09, 0.02)	0.275	−0.02 (−0.07, 0.04)	0.01 (−0.05, 0.06)	0.00 (−0.06, 0.05)	0.831	0.03 (−0.03, 0.08)	−0.03 (−0.08, 0.03)	−0.02 (−0.07, 0.04)	0.292

The authors apologize for any inconvenience caused to the readers by the changes, stating it does not affect the scientific results. The original manuscript will remain online on the article webpage, with a reference to this Erratum.
